# 3-Hydr­oxy-5,5-dimethyl-2-(2-oxo­propyl)cyclo­hex-2-enone

**DOI:** 10.1107/S1600536809049046

**Published:** 2009-11-25

**Authors:** Roberto Martínez, Simón Hernández-Ortega, Liz Triana, Jose Camacho

**Affiliations:** a Instituto de Química, Universidad Nacional Autónoma de México, Circuito Exterior, Ciudad Universitaria, México 04510, Mexico; b Laboratorio 223, Departamento de Química, Universidad Simón Bolívar (USB), Apartado 47206, Caracas 1080-A, Venezuela

## Abstract

The title compound, C_11_H_16_O_3_, was obtained by reaction of dimedone, 5,5-dimethylcyclohexane-1,3-dione, and α-chloro­acetone. The cyclo­hexenone ring exhibits an envelope conformation with puckering amplitudes *Q* = 0.433 (2) and Φ = −109.0 (3)°. The 2-oxopropyl fragment is almost perpendicular to the cyclo­hexa­none ring [dihedral angle = 77.72 (8)°]. In the crystal, the mol­ecules are linked to each other through O—H⋯O hydrogen bonding, building a chain parallel to the *b* axis.

## Related literature

The title compound is used in the synthesis of heterocyclic compounds. For the general synthesis of various heterocyclic compounds, see: Knorr (1884 ▶); Paal (1885 ▶); Martínez *et al.* (1995 ▶, 2002 ▶, 2006 ▶). For related structures, see: Nagarajan *et al.* (1986 ▶); Schaeffer & Vince (1962 ▶); Selvanayagam *et al.* (2003 ▶). For puckering parameters, see: Cremer & Pople (1975 ▶).
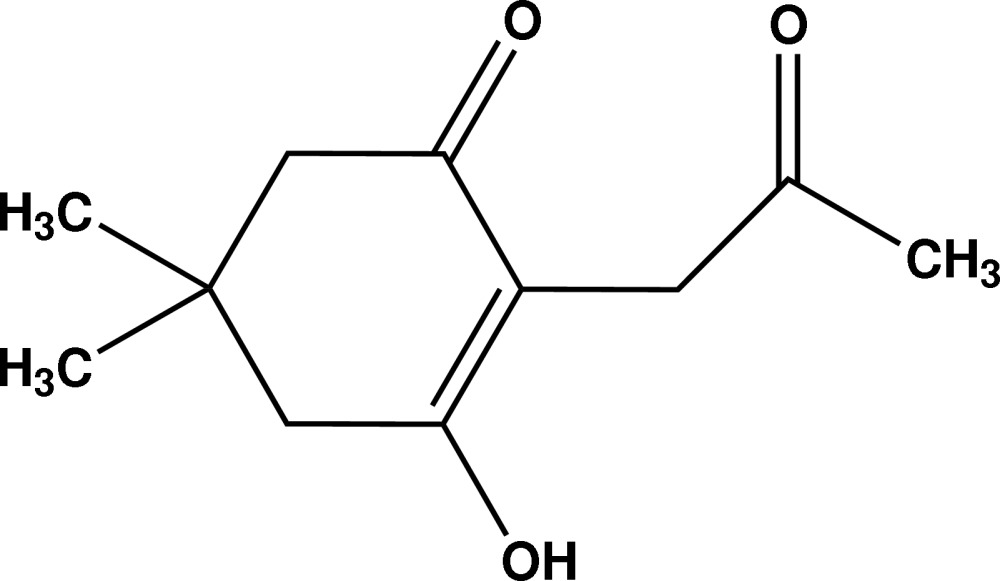


## Experimental

### 

#### Crystal data

C_11_H_16_O_3_
*M* *_r_* = 196.24
Monoclinic, 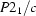

*a* = 10.005 (3) Å
*b* = 13.633 (4) Å
*c* = 8.441 (2) Å
β = 105.352 (4)°
*V* = 1110.3 (5) Å^3^
*Z* = 4
Mo *K*α radiation
μ = 0.08 mm^−1^
*T* = 298 K
0.32 × 0.16 × 0.15 mm

#### Data collection

Bruker SMART CCD area-detector diffractometer
Absorption correction: none
8977 measured reflections
2032 independent reflections
1573 reflections with *I* > 2σ(*I*)
*R*_int_ = 0.044

#### Refinement

*R*[*F* ^2^ > 2σ(*F* ^2^)] = 0.045
*wR*(*F* ^2^) = 0.126
*S* = 1.06
2032 reflections
134 parameters
1 restraint
H atoms treated by a mixture of independent and constrained refinement
Δρ_max_ = 0.22 e Å^−3^
Δρ_min_ = −0.19 e Å^−3^

### 

Data collection: *SMART* (Bruker, 1999 ▶); cell refinement: *SAINT* (Bruker, 1999 ▶); data reduction: *SAINT*; program(s) used to solve structure: *SHELXTL* (Sheldrick, 2008 ▶); program(s) used to refine structure: *SHELXTL*; molecular graphics: *ORTEPIII* (Burnett & Johnson, 1996 ▶), *ORTEP-3 for Windows* (Farrugia, 1997 ▶) and *PLATON* (Spek, 2009 ▶); software used to prepare material for publication: *SHELXTL*.

## Supplementary Material

Crystal structure: contains datablocks I, global. DOI: 10.1107/S1600536809049046/dn2511sup1.cif

Structure factors: contains datablocks I. DOI: 10.1107/S1600536809049046/dn2511Isup2.hkl

Additional supplementary materials:  crystallographic information; 3D view; checkCIF report

## Figures and Tables

**Table 1:** Hydrogen-bond geometry (Å, °)

*D*—H⋯*A*	*D*—H	H⋯*A*	*D*⋯*A*	*D*—H⋯*A*
O2—H2⋯O1^i^	0.877 (14)	1.692 (14)	2.5685 (16)	176.9 (18)

Symmetry code: (i) 
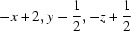
.

**Fig. 1.**
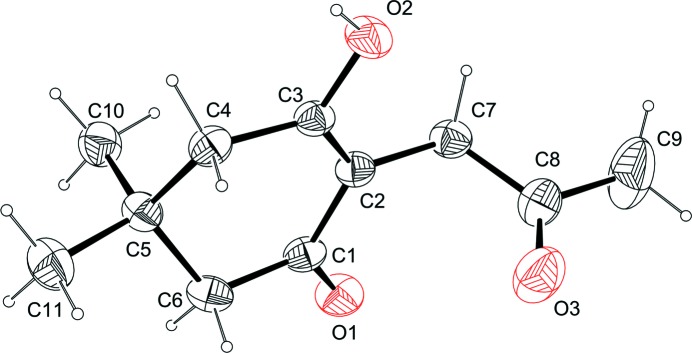
Molecular structure of (I) with the atom labeling scheme. Ellipsoids are drawn at the 30% probability level. H atoms are represented as small spheres of arbitrary radii.

**Fig. 2.**
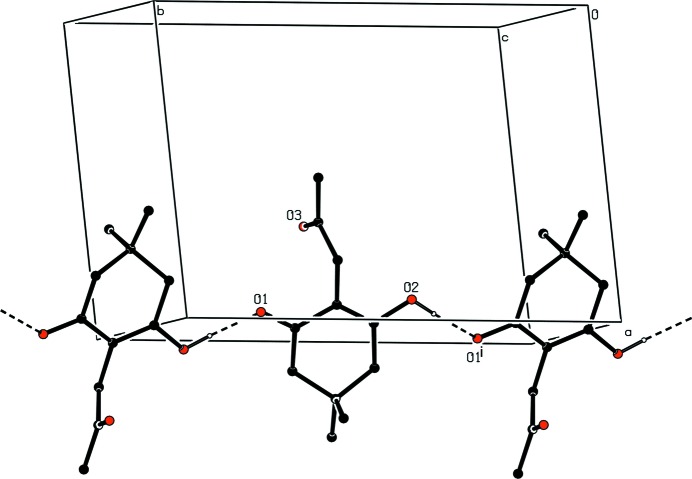
Partial packing view showing the formation of infinite chains parallel to the b axis through O-H···O hydrogen bonds. H atoms not involved in hydrogen bondings have been omitted for clarity. H bonds are shown as dashed lines. [Symmetry codes: (i) -x+2, y-1/2, -z+1/2]

## supplementary crystallographic information

### Comment

1,4-dicarbonyl derivatives are important intermediates in organic chemistry, they have great utility in the synthesis of various heterocyclic compounds. The Paal-Knorr reaction (Knorr, 1884; Paal, 1885) uses 1,4-dicarbonyl compounds to obtain different types of molecules like pyrroles, furans or thiophenes. (Martínez *et al.*, 1995, 2002, 2006).

In the title compound, The cyclohexenone ring adopt an envelope conformation with overall puckering amplitudes *Q* 0.433 (2) and Φ = -109.0 (3) (Cremer & Pople, 1975), with the keto-enol (O1—C1—C2—C3—O2) fragment planar and the acetonyl moiety is almost perpendicular to this plane making a dihedral angle of 77.72 (8) ° (Fig. 1). Distances and angles agree with values reported in related compounds ( Nagarajan *et al.*, 1986; Schaeffer & Vince, 1962; Selvanayagam, *et al.*, 2003)

In the crystal the molecules are linked to each other through O-H···O hydrogen bonding building a chain parallel to the b axis (Table 1).

### Experimental

A slurry of dimedone (0.01 equiv), chloroketone (0.01 equiv), and anhydrous potassium carbonate (0.01 equiv) in chloroform was kept stirred at room temperature for 48 h. The mixture was filtered; the insoluble salts were dissolved in water and the filtered solution was made acidic with concentrated HCl. The precipitate was filtered off and washed with water. Yield 70%. The Melting point (uncorrected) was determined on a Melt-Tem II melting points apparatus: 406–407 K. (Martínez *et al.*, 2006). The title compound (I) was obtained as suitable crystal for X-ray analysis after recrystallization of the solid from 1:1 Methanol-Ethyl Acetate mixture. ^1^H NMR [200 MHz, CDCl_3_, δ (p.p.m.)]: 9.0 (brs, 1H), 3.41 (s, 2H), 2.35 (s, 4H), 2.16 (s, 3H), 1.08 (s, 6H).

### Refinement

H atom on hydroxyl group was found in Fourier map and refined with *U*_iso_(H) = 1.2 UeqC(O). H on C atoms were placed in geometrically idealized positions [0.97 Å(CH_2_) and 0.96 Å (CH_3_)] and treated as riding on their parent atom with *U*_iso_(H) = 1.2 UeqC(CH_2_) and 1.5 UeqC(CH3).

### Crystal data

**Table:**

C_11_H_16_O_3_	*F*(000) = 424
*M**_r_* = 196.24	*D*_x_ = 1.174 Mg m^−^^3^
Monoclinic, *P*2_1_/*c*	Melting point: 406 K
Hall symbol: -P 2ybc	Mo *K*α radiation, λ = 0.71073 Å
*a* = 10.005 (3) Å	Cell parameters from 4286 reflections
*b* = 13.633 (4) Å	θ = 2.5–25.3°
*c* = 8.441 (2) Å	µ = 0.08 mm^−^^1^
β = 105.352 (4)°	*T* = 298 K
*V* = 1110.3 (5) Å^3^	Prism, colourless
*Z* = 4	0.32 × 0.16 × 0.15 mm

### Data collection

**Table:**

Bruker SMART CCD area-detector diffractometer	1573 reflections with *I* > 2σ(*I*)
Radiation source: fine-focus sealed tube	*R*_int_ = 0.044
graphite	θ_max_ = 25.4°, θ_min_ = 2.6°
Detector resolution: 0.83 pixels mm^-1^	*h* = −12→12
ω scans	*k* = −16→16
8977 measured reflections	*l* = −10→10
2032 independent reflections	

### Refinement

**Table:**

Refinement on *F*^2^	Secondary atom site location: difference Fourier map
Least-squares matrix: full	Hydrogen site location: inferred from neighbouring sites
*R*[*F*^2^ > 2σ(*F*^2^)] = 0.045	H atoms treated by a mixture of independent and constrained refinement
*wR*(*F*^2^) = 0.126	*w* = 1/[σ^2^(*F*_o_^2^) + (0.0704*P*)^2^ + 0.0505*P*] where *P* = (*F*_o_^2^ + 2*F*_c_^2^)/3
*S* = 1.06	(Δ/σ)_max_ = 0.001
2032 reflections	Δρ_max_ = 0.22 e Å^−^^3^
134 parameters	Δρ_min_ = −0.19 e Å^−^^3^
1 restraint	Extinction correction: *SHELXTL* (Sheldrick, 2008), Fc^*^=kFc[1+0.001xFc^2^λ^3^/sin(2θ)]^-1/4^
Primary atom site location: structure-invariant direct methods	Extinction coefficient: 0.021 (5)

### Special details

**Table:**

Geometry. All e.s.d.'s (except the e.s.d. in the dihedral angle between two l.s. planes) are estimated using the full covariance matrix. The cell e.s.d.'s are taken into account individually in the estimation of e.s.d.'s in distances, angles and torsion angles; correlations between e.s.d.'s in cell parameters are only used when they are defined by crystal symmetry. An approximate (isotropic) treatment of cell e.s.d.'s is used for estimating e.s.d.'s involving l.s. planes.
Refinement. Refinement of *F*^2^ against ALL reflections. The weighted *R*-factor *wR* and goodness of fit *S* are based on *F*^2^, conventional *R*-factors *R* are based on *F*, with *F* set to zero for negative *F*^2^. The threshold expression of *F*^2^ > σ(*F*^2^) is used only for calculating *R*-factors(gt) *etc*. and is not relevant to the choice of reflections for refinement. *R*-factors based on *F*^2^ are statistically about twice as large as those based on *F*, and *R*- factors based on ALL data will be even larger.

### Fractional atomic coordinates and isotropic or equivalent isotropic displacement parameters (Å^2^)

**Table:**

	*x*	*y*	*z*	*U*_iso_*/*U*_eq_	
O1	0.96277 (13)	0.77843 (7)	0.23295 (14)	0.0636 (4)	
O2	0.92396 (12)	0.44317 (7)	0.16061 (14)	0.0577 (4)	
H2	0.9653 (18)	0.3880 (11)	0.199 (2)	0.069*	
O3	0.68935 (15)	0.65312 (12)	0.26209 (17)	0.0924 (5)	
C1	1.00978 (16)	0.69523 (9)	0.27353 (17)	0.0457 (4)	
C2	0.94081 (15)	0.61103 (9)	0.18949 (17)	0.0431 (4)	
C3	0.99433 (15)	0.52082 (9)	0.23391 (17)	0.0422 (4)	
C4	1.12691 (15)	0.50283 (10)	0.36178 (18)	0.0467 (4)	
H4A	1.1757	0.4495	0.3255	0.056*	
H4B	1.1055	0.4820	0.4622	0.056*	
C5	1.22308 (15)	0.59193 (10)	0.39985 (17)	0.0473 (4)	
C6	1.13588 (17)	0.68191 (11)	0.41453 (18)	0.0529 (4)	
H6A	1.1069	0.6766	0.5151	0.064*	
H6B	1.1935	0.7399	0.4234	0.064*	
C7	0.80924 (16)	0.62619 (11)	0.05840 (18)	0.0510 (4)	
H7A	0.8230	0.6794	−0.0119	0.061*	
H7B	0.7901	0.5674	−0.0085	0.061*	
C8	0.68542 (18)	0.64907 (12)	0.1183 (2)	0.0616 (5)	
C9	0.5541 (2)	0.6657 (2)	−0.0119 (3)	0.1196 (10)	
H9A	0.5341	0.6094	−0.0824	0.179*	
H9B	0.5639	0.7224	−0.0754	0.179*	
H9C	0.4796	0.6762	0.0382	0.179*	
C10	1.29416 (18)	0.60741 (12)	0.26238 (19)	0.0609 (5)	
H10A	1.2252	0.6147	0.1596	0.091*	
H10B	1.3516	0.5518	0.2568	0.091*	
H10C	1.3503	0.6655	0.2844	0.091*	
C11	1.33259 (19)	0.57556 (14)	0.5624 (2)	0.0702 (5)	
H11A	1.3837	0.5168	0.5555	0.105*	
H11B	1.2880	0.5691	0.6496	0.105*	
H11C	1.3948	0.6305	0.5844	0.105*	

### Atomic displacement parameters (Å^2^)

**Table:**

	*U*^11^	*U*^22^	*U*^33^	*U*^12^	*U*^13^	*U*^23^
O1	0.0798 (8)	0.0312 (6)	0.0775 (8)	0.0051 (5)	0.0171 (6)	0.0014 (5)
O2	0.0632 (8)	0.0335 (6)	0.0708 (7)	−0.0047 (5)	0.0083 (6)	−0.0065 (5)
O3	0.0792 (10)	0.1301 (13)	0.0747 (9)	0.0173 (9)	0.0326 (8)	0.0072 (8)
C1	0.0586 (9)	0.0313 (7)	0.0510 (8)	0.0022 (6)	0.0213 (7)	−0.0002 (6)
C2	0.0489 (9)	0.0356 (7)	0.0451 (8)	0.0023 (6)	0.0130 (7)	−0.0011 (6)
C3	0.0486 (8)	0.0330 (7)	0.0474 (8)	−0.0033 (6)	0.0171 (7)	−0.0039 (6)
C4	0.0524 (9)	0.0362 (7)	0.0527 (8)	0.0045 (6)	0.0162 (7)	0.0032 (6)
C5	0.0493 (9)	0.0441 (8)	0.0460 (8)	−0.0033 (6)	0.0086 (7)	−0.0012 (6)
C6	0.0665 (11)	0.0404 (8)	0.0509 (8)	−0.0057 (7)	0.0136 (8)	−0.0086 (6)
C7	0.0573 (10)	0.0442 (8)	0.0496 (8)	0.0046 (7)	0.0107 (7)	0.0000 (6)
C8	0.0595 (11)	0.0612 (10)	0.0649 (11)	0.0083 (8)	0.0183 (9)	0.0112 (8)
C9	0.0639 (14)	0.193 (3)	0.0988 (17)	0.0375 (16)	0.0157 (13)	0.0363 (18)
C10	0.0557 (10)	0.0657 (11)	0.0631 (10)	−0.0091 (8)	0.0188 (8)	−0.0028 (8)
C11	0.0639 (11)	0.0764 (12)	0.0604 (10)	−0.0016 (9)	−0.0008 (9)	0.0026 (9)

### Geometric parameters (Å, °)

**Table:**

O1—C1	1.2409 (16)	C6—H6A	0.9700
O2—C3	1.3298 (16)	C6—H6B	0.9700
O2—H2	0.877 (14)	C7—C8	1.490 (2)
O3—C8	1.2050 (19)	C7—H7A	0.9700
C1—C2	1.4276 (19)	C7—H7B	0.9700
C1—C6	1.499 (2)	C8—C9	1.491 (3)
C2—C3	1.3541 (18)	C9—H9A	0.9600
C2—C7	1.493 (2)	C9—H9B	0.9600
C3—C4	1.492 (2)	C9—H9C	0.9600
C4—C5	1.530 (2)	C10—H10A	0.9600
C4—H4A	0.9700	C10—H10B	0.9600
C4—H4B	0.9700	C10—H10C	0.9600
C5—C10	1.528 (2)	C11—H11A	0.9600
C5—C11	1.529 (2)	C11—H11B	0.9600
C5—C6	1.529 (2)	C11—H11C	0.9600
			
C3—O2—H2	111.8 (12)	C8—C7—C2	115.25 (13)
O1—C1—C2	119.99 (14)	C8—C7—H7A	108.5
O1—C1—C6	120.52 (13)	C2—C7—H7A	108.5
C2—C1—C6	119.45 (12)	C8—C7—H7B	108.5
C3—C2—C1	119.28 (13)	C2—C7—H7B	108.5
C3—C2—C7	122.51 (12)	H7A—C7—H7B	107.5
C1—C2—C7	118.19 (12)	O3—C8—C7	122.90 (16)
O2—C3—C2	118.19 (13)	O3—C8—C9	121.54 (17)
O2—C3—C4	117.74 (12)	C7—C8—C9	115.56 (16)
C2—C3—C4	124.07 (12)	C8—C9—H9A	109.5
C3—C4—C5	114.25 (11)	C8—C9—H9B	109.5
C3—C4—H4A	108.7	H9A—C9—H9B	109.5
C5—C4—H4A	108.7	C8—C9—H9C	109.5
C3—C4—H4B	108.7	H9A—C9—H9C	109.5
C5—C4—H4B	108.7	H9B—C9—H9C	109.5
H4A—C4—H4B	107.6	C5—C10—H10A	109.5
C10—C5—C11	109.57 (14)	C5—C10—H10B	109.5
C10—C5—C6	109.94 (12)	H10A—C10—H10B	109.5
C11—C5—C6	109.45 (12)	C5—C10—H10C	109.5
C10—C5—C4	110.09 (12)	H10A—C10—H10C	109.5
C11—C5—C4	109.51 (13)	H10B—C10—H10C	109.5
C6—C5—C4	108.26 (12)	C5—C11—H11A	109.5
C1—C6—C5	114.28 (11)	C5—C11—H11B	109.5
C1—C6—H6A	108.7	H11A—C11—H11B	109.5
C5—C6—H6A	108.7	C5—C11—H11C	109.5
C1—C6—H6B	108.7	H11A—C11—H11C	109.5
C5—C6—H6B	108.7	H11B—C11—H11C	109.5
H6A—C6—H6B	107.6		
			
O1—C1—C2—C3	179.22 (13)	C3—C4—C5—C11	163.65 (13)
C6—C1—C2—C3	−3.0 (2)	C3—C4—C5—C6	44.38 (16)
O1—C1—C2—C7	−2.5 (2)	O1—C1—C6—C5	−150.70 (14)
C6—C1—C2—C7	175.23 (13)	C2—C1—C6—C5	31.5 (2)
C1—C2—C3—O2	176.08 (12)	C10—C5—C6—C1	69.73 (17)
C7—C2—C3—O2	−2.1 (2)	C11—C5—C6—C1	−169.88 (13)
C1—C2—C3—C4	−3.2 (2)	C4—C5—C6—C1	−50.56 (17)
C7—C2—C3—C4	178.68 (13)	C3—C2—C7—C8	102.90 (17)
O2—C3—C4—C5	161.35 (13)	C1—C2—C7—C8	−75.28 (18)
C2—C3—C4—C5	−19.4 (2)	C2—C7—C8—O3	−1.6 (2)
C3—C4—C5—C10	−75.82 (16)	C2—C7—C8—C9	179.14 (19)

### Hydrogen-bond geometry (Å, °)

**Table:**

*D*—H···*A*	*D*—H	H···*A*	*D*···*A*	*D*—H···*A*
O2—H2···O1^i^	0.88 (1)	1.69 (1)	2.5685 (16)	177 (2)

Symmetry codes: (i) −*x*+2, *y*−1/2, −*z*+1/2.
